# Managing insect and plant pathogen pests with organic and conventional pesticides in onions

**DOI:** 10.1093/jee/toad201

**Published:** 2023-11-01

**Authors:** Natalie Constancio, Douglas Higgins, Mary Hausbeck, Zsofia Szendrei

**Affiliations:** Department of Entomology, Michigan State University, East Lansing, MI 48824, USA; Eastern Shore Agriculture Research and Extension Center, Virginia Polytechnic Institute and State University, Painter, VA 23350, USA; Department of Plant, Soil, and Microbial Sciences, Michigan State University, East Lansing, MI 48824, USA; Department of Entomology, Michigan State University, East Lansing, MI 48824, USA

**Keywords:** action threshold, spinosyn, plant disease, onion thrips, insecticide

## Abstract

Onion thrips (*Thrips tabaci* Lindeman, Thysanoptera: Thripidae) is a significant insect pest of onions (*Allium cepa* L., Asparagales: Amaryllidaceae). In addition to feeding on onion foliage, they may spread plant pathogens. Currently, onion thrips and pathogens are managed as separate pests with insecticides and fungicides. It may be beneficial to manage these pests simultaneously as limiting onion thrips may reduce pathogen damage. We tested combinations of bio- and conventional pesticides in a season-long management program in Michigan onion fields. From 2020 to 2022, we counted onion thrips weekly and visually estimated plant foliage necrotic damage (%) in experimental plots each year. In 2020, we tested 6 treatment programs including: azadirachtin, spinosad, a copper-based fungicide, azadirachtin + copper-based fungicide, spinosad + copper-based fungicide, and untreated control. The thrips populations were not significantly reduced compared to the control, but necrotic damage was reduced significantly in spinosad-treated plots. In 2021, we tested a combination of 8 bio- and conventional pesticide programs. Compared to the control, the bioinsecticides did not reduce onion thrips populations, but the conventional pesticide programs reduced both onion thrips numbers and necrotic damage. In 2022, we tested only conventional insecticide programs but included 3 different action thresholds for initiation and applied them with or without a fungicide, for 8 treatments. All insecticide programs reduced onion thrips compared to the control, the action threshold did not impact thrips numbers significantly. Overall, the use of action thresholds can lead to fewer insecticide applications and a lower incidence of leaf damage.

## Introduction

Overreliance on pesticides in agriculture has numerous negative effects related to public health, non-target impacts, and pesticide resistance (Pimentel 2005, Rolim et al. 2020, Serrão et al. 2022, Silva et al. 2020). Despite these negative impacts, pesticides continue to be one of the most effective tools used in integrated pest management (IPM) to control pests (Reddy 2016), and the incorporation of biopesticides into conventional agricultural practices could lead to greater sustainability. In organic agriculture, pesticides are limited to those approved by the Organic Materials Review Institute and can only be used if all other management options have been exhausted (USDA 2023). The sustainable use of pesticides in both conventional and organic management systems relies on strategies that limit their use. These strategies include the use of action thresholds, a thorough understanding of the biology and ecology of the system, the correct timing and dose of applications, and the use of appropriate active ingredients for specific pest species (Siegwart et al. 2015).

The complexities of pest management are exacerbated when different pest species co-occur and affect each other on the host plant. Management tactics that focus on a single pest may compound other pest problems, or in some instances, the suppression of one pest leads to a decrease in another. When plant pathogens are transmitted by insect pests, controlling the insect may reduce pathogen symptoms because insect feeding can provide entry points for plant pathogens (McKenzie et al. 1993, Grode et al. 2017, 2019, Leach et al. 2017). Action thresholds developed for one pest may not apply to multiple interacting pests and thresholds may need to be revised to optimize management.

Farmers are facing new pest management challenges, including the overall rising costs of pesticides, partially due to increases in the cost of development of pesticides that favor highly selective products, which are safer for non-target organisms (Sparks 2013). Overall, there are few active ingredients to control insects, increasing the need to understand the biology of the pest complex (Sparks 2013). Prophylactic applications of insecticide are increasingly unsustainable and action thresholds are a critical part of many insect management programs (Anonymous 2001, Leach et al. 2017, 2019, Ternest et al. 2020). Farmers are increasingly interested in incorporating IPM tools to reduce reliance on pesticides and delay pest resistance (Leach et al. 2017, Iglesias et al. 2021b). Generally, biopesticides are short-lived and more effective against immature than adult insects (Golec et al. 2020). If biopesticides are to be adopted more widely in agriculture, their efficacy and application timing must be carefully evaluated as they do not perform well when insect population pressure is high (Dively et al. 2020).

Onion thrips (*Thrips tabaci* Lindeman, Thysanoptera: Thripidae) is a significant insect pest of onions, (*Allium cepa* L., Asparagales: Amaryllidaceae), and if not controlled, can result in severe crop loss (Lewis 1991, Fournier et al. 1995, Diaz-Montano et al. 2011, Gill et al. 2015). Onion thrips are resistant to several classes of insecticides; rotating active ingredients and using action thresholds sustain insecticide efficacy (Martin et al. 2003, Shelton et al. 2006, Nault and Shelton 2010, Diaz-Montano et al. 2011, Leach et al. 2019, Adesanya et al. 2020). One of the most effective insecticides for onion thrips control is spinetoram (Gill et al. 2015, Moretti et al. 2019). Onion growers consider it a high priority to maintain its efficacy (Leach et al. 2017, 2019). Despite efforts to adopt IPM strategies for onion thrips management, insecticides remain the cornerstone for management (Leach et al. 2017, 2020, Devi and Roy 2018, Yadav et al. 2018, Iglesias et al. 2021a, b). Among the bioinsecticides, spinosad is the most effective for onion thrips control (Nault and Hessney 2005, Dively et al. 2020, Iglesias et al. 2021a, b) and it has the same mode of action as spinetoram (spinosyns). Other biopesticides such as azadirachtin and pyrethrin have been tested for onion thrips control with and without adjuvants (Dively et al. 2020, Iglesias et al., 2021b)

Onion thrips damage the onion foliage by feeding and transmitting plant pathogens (Bhangale and Joi 1983, McKenzie et al. 1993, Dutta et al. 2014, Leach et al. 2017). Two important fungal pathogens of onion in Michigan include *Colletotrichum coccodes* (Wallr) (Glomerellales: Glomerellacaea), first identified in 2012 (Rodriguez-Salamanca et al. 2012), and *Stemphylium vesicarium* (E.G. Simmons) (Pleosporales: Pleosporaceae), a destructive pathogen of the eastern production region (Hausbeck and Werling 2018, Hay et al. 2022). The relationship between facultative onion pathogens and onion thrips has been the focus of various studies (Grode et al. 2017, 2019, Leach et al. 2020, Constancio et al. 2022) but the influence of bio- and conventional pesticides on onion foliage and yields is not well understood. Managing 2 organisms simultaneously may be complicated by tank mixing insecticides and fungicides which may reduce insecticide efficacy (Nault et al. 2013). The action thresholds used to control onion thrips (Nault and Shelton 2010) have been developed without considering the interaction between thrips and onion plant pathogens.

The goal of this study was to evaluate the efficacy of bio- and conventional pesticides in a season-long program to control multiple onion pests and develop a combined onion thrips and Stemphylium leaf blight management pesticide program, with resistance management practices in mind. Our first goal was to broaden the insecticide options currently used in onion thrips management by testing bioinsecticides. In 2020, we evaluated the efficacy of biopesticide programs by comparing 6 different treatments. Building off these results in 2021, we then compared 8 different bio- and conventional pesticide programs side-by-side. We repeated some of the biopesticide programs from 2020 and added 2 new conventional pesticide programs to address the differences in the efficacy of bio- and conventional pesticides. The conventional pesticide programs we evaluated in 2021 follow the recommended pesticide programs but are not appropriate for insecticide resistance management. Therefore, we made the decision to change pesticide programs to address the overarching goal of our study. In 2022, we tested varying thrips action thresholds of the conventional pesticide program from 2021. We did this to reduce the total number of applications of conventional pesticides and allow better coherence to insecticide resistance management guidelines. To measure the impact of our treatments on onion pests, we counted onion thrips per leaf weekly, and visually estimated leaf necrosis to measure foliar health.

## Materials and Methods

### Field Site

Experiments were conducted on muck soil, previously cropped to celery, from June 2020 to August 2022 on a commercial conventional onion farm located in Allegan County, MI. Onions were rotated to a new field every year and experimental plots were moved to a new location within the same farm each year. ‘Bradley’ onions were direct seeded in early April of each year on raised plant beds at a density of approximately 625,000 seeds/ha. Each plant bed was comprised of 8 rows spaced 0.15 m apart; onions were direct seeded 5 cm apart. Fertilization, herbicide application, and irrigation were managed by the grower cooperator, and weeds were removed manually to supplement commercial practices. Herbicide treatments were applied to the whole field by the grower using a broadcast sprayer prior to experimental applications. Treatment plots (6 m long × 1.5 m wide) were separated by a 0.6 m buffer and arranged in a randomized complete block design, with 4 replicates. All pesticides were applied as a foliar spray with a CO_2_ backpack sprayer and a broadcast boom. The boom was equipped with 3 XR8003 flat-fan nozzles (TeeJet Technologies, Wheaton, IL) spaced 45 cm apart, calibrated to 241 kPa to deliver 467 L/ha.

### Onion Thrips

Onion thrips counting began on 8 July 2020, 14 June 2021, and 14 June 2022 and included counting all thrips on 10 randomly selected plants per plot from the center 6 rows of the beds. Selected plants including the neck of the onion were visually assessed in a non-destructive manner for the total number of adult and larval onion thrips. Thrips were counted weekly until 27 July 2020, 9 August 2021, and 8 August 2022. In addition to recording onion thrips numbers, the number of leaves was counted weekly on 20 plants randomly selected from the center 6 rows of the field plots.

### Necrotic Plant Tissue

Necrotic plant tissue was assessed by visually estimating the percentage of necrotic tissue (0%–100%) per plot on 28 July and 3 and 10 August 2020; 23 and 30 July and 6 and 17 August 2021. In 2022, plots were assessed weekly from 20 June to 29 August, for a total of 10 assessments except for the first week of August. If there were signs of Stemphylium leaf blight, the pathogen was isolated from 50 plants and identified based on conidial morphology (Woudenberg et al. 2017).

### Pesticide Treatments

Treatments in 2020 included: (i) untreated control, (ii) azadirachtin (neem oil), (iii) spinosad (Entrust SC), (iv) azadirachtin + copper (Kocide 3000), (v) spinosad + copper, and (vi) copper (Table 1). Pesticides were applied weekly from 9 July until 4 August, and were tank mixed with a nonionic surfactant (Dyne-Amic, 0.05% v/v), for a total of 5 pesticide applications.

**Table 1.** Pesticides used in the onion thrips and onion disease management field experiments, 2020–2022. Group number refers to the mode of action of the pesticide.

Group #	Product name	Manufacturer	Active ingredient	Rate	Chemical type	OMRI^a^ certified	Year applied
UN	Neem oil	Platonix	Azadirachtin	7.8 ml/L	Insecticide	Yes	2020
UN	Neemix 4.5	Certus Biologicals	Azadirachtin	7.8 ml/L	Insecticide	Yes	2021
5	Entrust SC	Corteva	Spinosad	1.1 ml/L	Insecticide	Yes	2020, 2021
	Kocide 3000-O	DuPont	Copper hydroxide	3.6 ml/L	Bactericide/Fungicide	Yes	2020, 2021
23	Movento	Bayer CropScience	Spirotetramat	0.78 ml/L	Insecticide	No	2021, 2022
6, 28	Minecto Pro	Syngenta	Abamectin, Cyantraniliprole	1.5 ml/L	Insecticide	No	2021, 2022
5	Radiant SC	Corteva	Spinetoram	1.5 ml/L	Insecticide	No	2021, 2022
1	Lannate LV	Corteva	Methomyl	7.5 ml/L	Insecticide	No	2021
3	Warrior II	Syngenta	Lambda-cyhalothrin	0.3 ml/L	Insecticide	No	2021
6	Agri-Mek	Syngenta	Abamectin	0.5 ml/L	Insecticide	No	2021
7, 12	Miravis Prime	Syngenta	Pydiflumetofen, Fludioxonil	1.8 ml/L	Fungicide	No	2021, 2022
M5	Bravo Weather Stik	Adama Agricultural Solutions	Chlorothalonil	3.8 ml/L	Fungicide	No	2021, 2022
	Dyne-Amic 90	Helena Chemical Company	Methylated seed oils Organosilicone non-ionic surfactant	0.05% v/v	Surfactant	No	2020
	Activator 90	Loveland	Non-ionic surfactant	0.05% v/v	Surfactant	No	2021
	Syl-Tac	Wilbur-Ellis	Organosilicone surfactant Modified vegetable oil concentrate	0.10% v/v	Surfactant	No	2022

^a^OMRI, Organic Materials Review Institute.

Treatments in 2021 included: (i) untreated control, (ii) azadirachtin (Neemix 4.5), (iii) spinosad, (iv) azadirachtin + copper, (v) spinosad + copper, (vi) copper, (vii) conventional insecticide program + copper (CI + copper), and (viii) conventional insecticide and fungicide programs (CI + CF). All pesticides were tank-mixed with a nonionic surfactant (Activator 90, 0.05% v/v). Conventional insecticide treatments 7 and 8 contained the following applied twice, 7 days apart before rotating to the next insecticide: spirotetramat (Movento), abamectin + cyantraniliprole (MinectoPro), spinetoram (Radiant SC), methomyl + lambda-cyhalothrin (Lannate LV + Warrior II), and a single application of abamectin (Agri-Mek) (Table 1). The conventional fungicide program consisted of pydiflumetofen + fludioxonil (Miravis Prime) and chlorothalonil (Bravo Weather Stik), applied 7 days apart and rotated weekly for 6 weeks, before applying chlorothalonil. All pesticide programs began on 15 June and continued until 10 August.

In 2022, pesticide treatments were applied according to various thrips action thresholds and included: (i) untreated control, (ii) fungicide only, (iii) low threshold + fungicide, (iv) moderate threshold + fungicide, (v) high threshold + fungicide, (vi) low threshold, (vii) moderate threshold, and (viii) high threshold. Pesticides were tank mixed with an organosilicone surfactant (Syl-Tac, 1% v/v). All programs containing fungicides alternated between pydiflumetofen + fludioxonil and chlorothalonil weekly, starting on 5 July. The low action threshold was 0.5 thrips/leaf (6 applications) and included: spirotetramat, abamectin + cyantraniliprole, and spinetoram; each applied twice, 7 days apart. Spirotetramat’s recommended action threshold for onion thrips is 0.6 onion thrips per leaf, therefore, to test the efficacy of the low threshold and to ensure all 6 pesticide applications could be applied to the field, we reduced the action threshold to 0.5 onion thrips per leaf. The moderate threshold was 0.6 onion thrips/leaf (4 applications) and included abamectin + cyantraniliprole and spinetoram; each applied twice, 7 days apart. Abamectin + cyantraniliprole is typically the second product used in onion thrips management (applied after spirotetramat); however, the moderate threshold did not receive a spirotetramat application, so abamectin + cyantraniliprole was applied using the action threshold commonly recommended for spirotetramat. The high threshold was 1.0 thrips/leaf (2 applications) and included spinetoram applied 7 days apart. The low, moderate, and high threshold programs began on 5 July, 26 July, and 9 August, respectively.

### Data Analysis

Data from each year were analyzed separately. To determine the effect of treatment on onion thrips populations and the estimated necrotic plant tissue, the mean number of onion thrips per leaf was log-transformed, and the estimated percent necrotic plant tissue was logit-transformed to meet the assumptions of an analysis of variance (ANOVA) and compared with the ‘lme4’ package (Bates et al. 2015) using treatment as the fixed factor and date and plot as a random factor. Tukey’s HSD test (α = 0.05) was used to determine the differences between treatment means using the ‘multcomp’ package (v1.4-22; Hothorn, Bretz and Westfall, 2023). All analyses were completed in R (R Core Team 2023).

## Results

### 2020

#### Onion thrips.

In the control treatment, onion thrips numbers averaged 9.4 per leaf and ranged from 7.6 to 6.9 onion thrips per leaf in the treated plots. Although numerically different, there were no statistically significant differences in onion thrips numbers among the treatments (*F* = 0.53, *df* = 5, 77.49, *P* = 0.76).

#### Necrotic plant tissue.

**Fig. 1.**
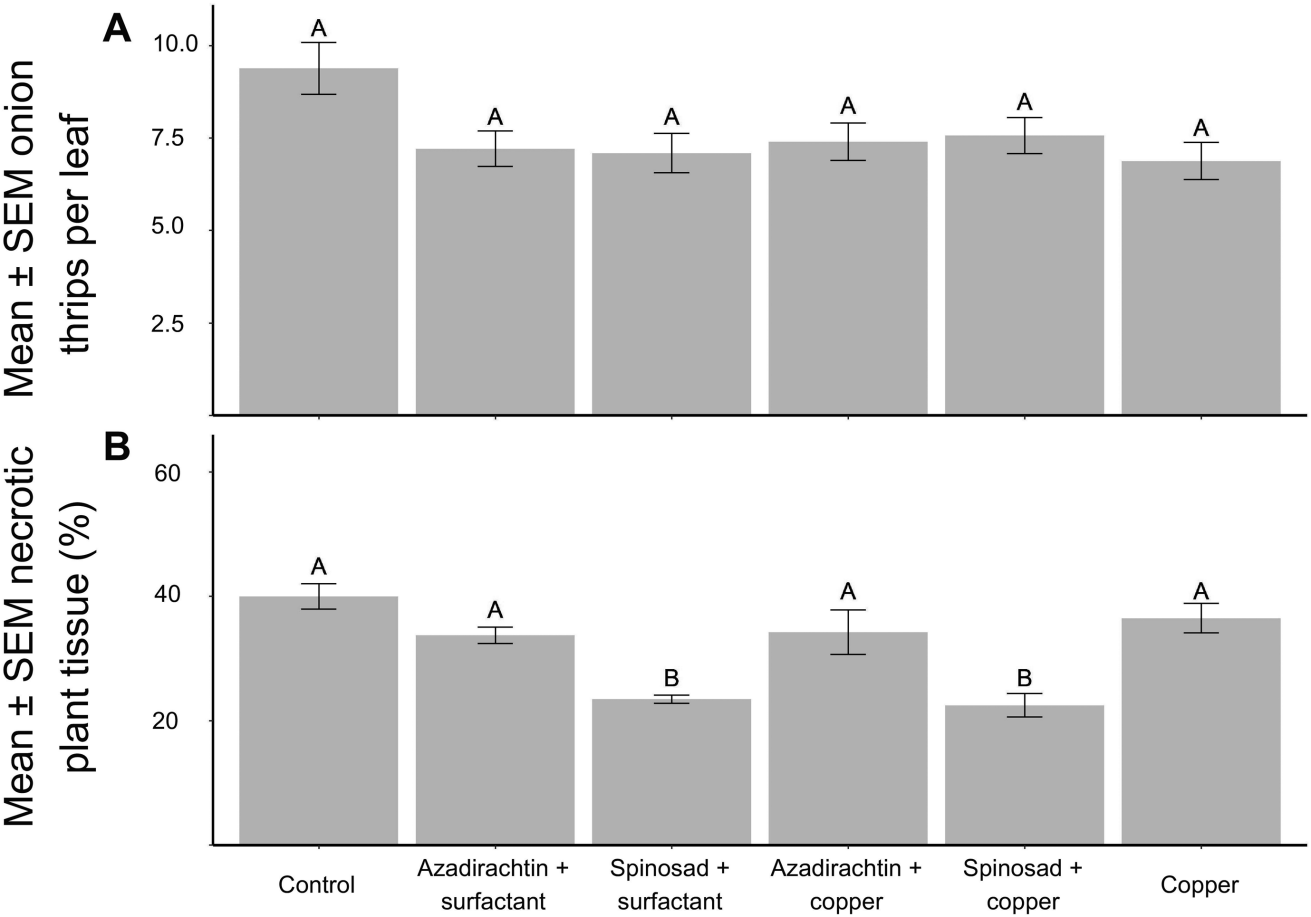
In 2020, pesticide treatments were applied weekly to an onion field. Each week, onion thrips were counted on 10 plants per plot and the % of necrotic tissue per plot was visually estimated. a) The seasonal mean ± SEM onion thrips per leaf for each treatment. b) The mean ± SEM estimated percent necrotic tissue on 3 August 2020. Different letters above the bars indicate significant differences (Tukey’s HSD, α = 0.05).

In adjacent experimental plots, the first plant with *Stemphylium*-like conidia was observed on 7 July. There was a significant effect of treatment on necrotic damage on 3 August 2020 (*F* = 12.33, *df* = 5, 15, *P* < 0.01, Fig. 1b; SupplementaryFig. S1). Compared to the control treatment, there was an approximately 20% reduction in necrosis in plots treated with spinosad (*t*-value = 5.73, *df* = 6, *P* < 0.01) and spinosad + copper (*t*-value = 6.09, *df* = 6, *P* < 0.01). There was no difference among the control, azadirachtin, azadirachtin + copper, and copper-only treatments.

### 2021

#### Onion thrips.

**Fig. 2.**
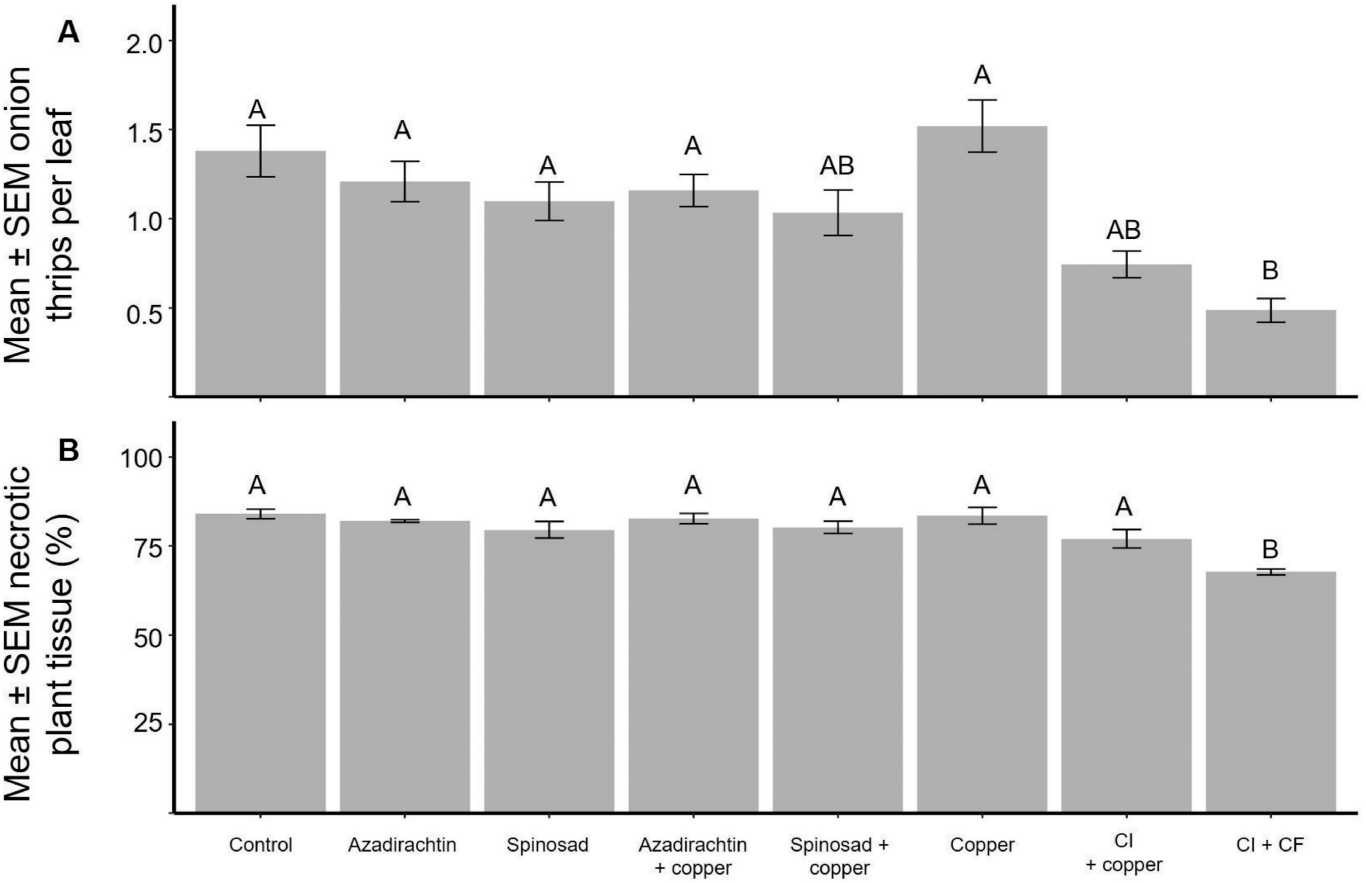
In 2021, 8 different pesticide treatments consisting of a mix of bio- and conventional pesticides were applied weekly in an onion field. Treatments labeled CI + copper represent the conventional insecticide program + copper fungicide, while the CI + CF treatment is the conventional insecticide program with a conventional fungicide program. Each week, onion thrips were counted on 10 plants per plot and the % necrotic tissue was visually estimated for each plot. a) The seasonal mean ± SEM onion thrips per leaf for each treatment. b) The mean ± SEM estimated percent necrotic tissue on 17 August 2021. Different letters above the bars indicate significant differences (Tukey’s HSD, α = 0.05).

Onion thrips numbers were significantly different among pesticide treatments (*F* = 5.34, *df* = 7, 27.85, *P* < 0.01; Fig. 2a). Compared to the control (1.4 onion thrips per leaf), there was no significant difference in the mean number of onion thrips per leaf in any of the biopesticide programs (ranging from 1.0 to 1.5 onion thrips per leaf). The conventional insecticide + fungicide program (0.5 onion thrips per leaf) reduced onion thrips numbers by 65% when compared to the control (*t*-value = 4.64, *df* = 72, *P* < 0.01). There was no statistical difference in onion thrips numbers between the conventional insecticide + copper treatment (0.7 onion thrips per leaf) and the conventional insecticide + fungicide program (*t*-value = −1.91, *df* = 72, *P* = 0.54).

#### Necrotic plant tissue.

In adjacent experimental plots, the first plant with Stemphylium-like conidia was observed on 24 June and the pathogen was isolated and identified from 50 plants. A moderate incidence of pink root rot and a low incidence (<10%) of anthracnose were also observed in adjacent experimental plots. We found that the estimated necrotic damage was significantly different among treatments on 17 August 2021 (*F* = 9.76, *df* = 7, 21, *P* < 0.01; Fig. 2b; SupplementaryFig. S2). Compared to all treatment programs, the fully conventional (insecticide + fungicide) program significantly reduced the amount of necrotic tissue in the field (*t*-value > 3.84, *df* = 8, *P* < 0.02). There were no significant differences in necrotic tissue among the other treatments.

### 2022

#### Onion thrips.

**Fig. 3.**
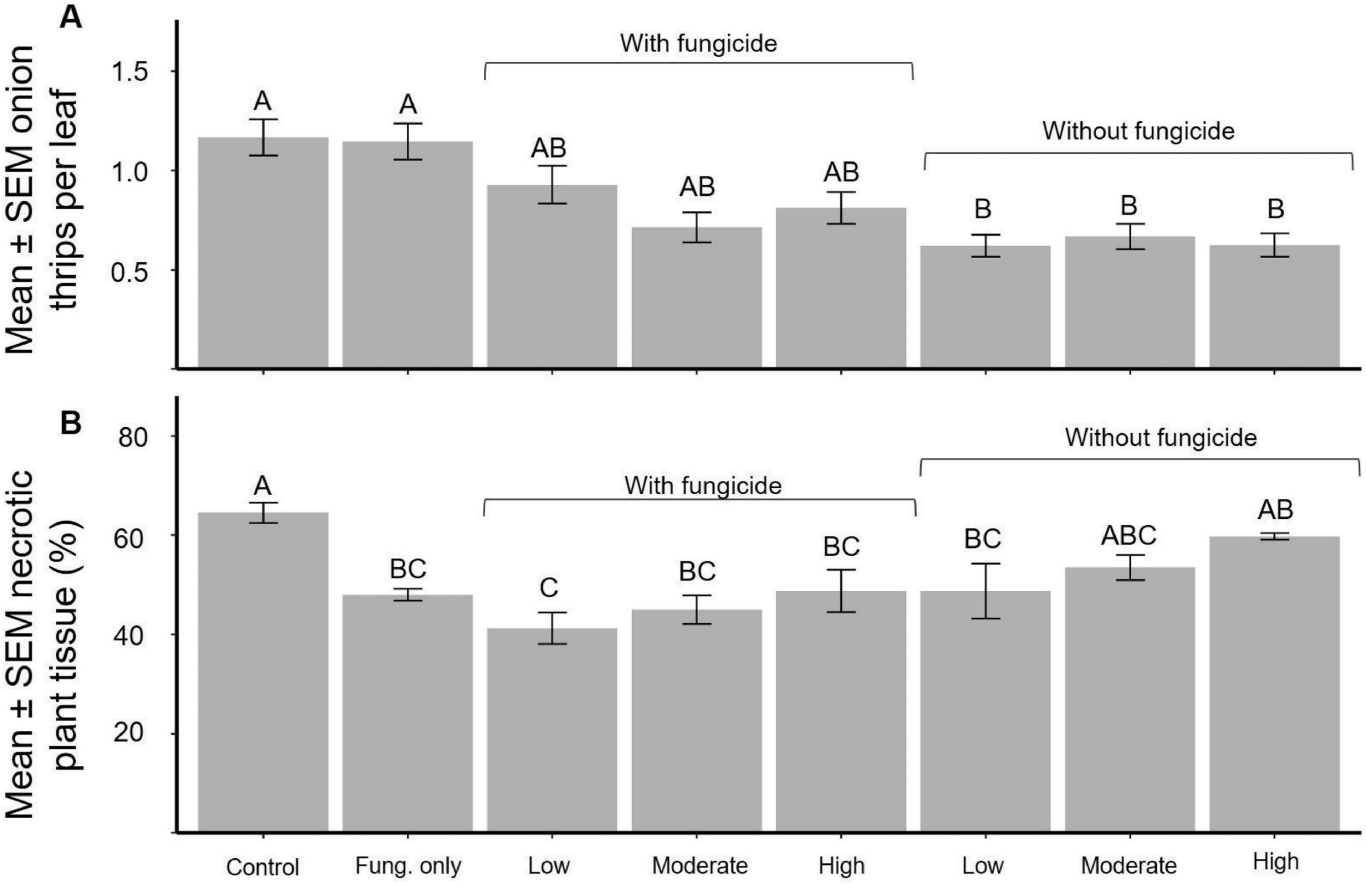
In 2022, 8 different conventional pesticide programs, with or without a fungicide, were applied to an onion field at 3 onion thrips action thresholds. Each week, onion thrips were counted on 10 plants per plot and the % necrotic tissue was visually estimated for each plot. Low threshold applications began when the onion thrips populations reached 0.5 onion thrips per leaf, the moderate threshold was 0.6 onion thrips per leaf, and the high threshold was 1.0 onion thrips per leaf. The fungicide-only treatments were applied weekly and began when the low onion thrips action threshold was reached. a) The seasonal mean ± SEM onion thrips per leaf for each treatment. b) The mean ± SEM estimated percent necrotic tissue on 29 August 2022. Different letters above the bars indicate significant differences (Tukey’s HSD, α = 0.05).

Overall, treatment had a significant effect on onion thrips numbers (*F* = 4.33, *df* = 7, 21, *P* < 0.01; Fig. 3a). Treatments with or without fungicides had similar numbers of onion thrips throughout the growing season (0.81 onion thrips per leaf vs. 0.63 onion thrips per leaf). All insecticide-only treatments, regardless of action threshold, reduced onion thrips populations by about 54% when compared to the control (1.2 onion thrips per leaf; *t*-value < −3.44, *df* = 56, *P* < 0.02). When comparing treatments across all insecticide programs, with or without fungicide, all of them reduced onion thrips similarly, regardless of the action threshold.

#### Necrotic plant tissue.

There was a significant effect of treatment on necrotic damage on 29 August (*F* = 5.93, *df* = 7, 24, *P* < 0.01; Fig. 3b; SupplementaryFig. S3). When compared to the control treatment the low action threshold + fungicide significantly reduced the amount of necrotic tissue (*t*-value = 5.2, *df* = 21, *P* < 0.01). Similarly, the low action threshold + fungicide treatment also significantly reduced necrotic tissue in the field when compared to the high action threshold without fungicide (*t*-value = −4.14, *df* = 21, *P* < 0.01). When compared to the control treatment, the moderate action threshold without a fungicide and the high action threshold without a fungicide did not reduce the amount of necrotic tissue.

## Discussion

Our results demonstrated that the bioinsecticides we tested cannot reduce onion thrips sufficiently to keep their populations below the commonly used action threshold of 1 thrips/leaf (Nault and Shelton, 2010) even under different pest pressures observed in 2020 (7–10 thrips/leaf) and 2021 (1–1.5 thrips/leaf). We expected the bioinsecticides to perform better in the low insect-pressure year (2021), but this was not the case. The 2021 growing season had nearly twice as much rainfall as 2020 (average rainfall June–August: 18.4 cm = 2020, 34.8 cm = 2021, MSU Enviroweather 2023), so bioinsecticides or onion thrips may have washed off the plants. Biopesticides are often more susceptible to extreme weather conditions than conventional pesticides (Gill et al. 2015, Fenibo et al. 2021, Fronk 2022). Our results represent the best-case scenario for the performance of biopesticides: there were 5 and 9 weekly applications in 2020 and 2021, respectively. These types of calendar-based applications are not representative of typical organic pest management programs, indicating that even these frequent applications were not able to provide sufficient thrips control.

Interestingly, azadirachtin and spinosad performed similarly in both years for thrips suppression relative to the control. Based on previous research, we expected that spinosad would perform significantly better than azadirachtin for onion thrips suppression (Dively et al. 2020, Iglesias et al. 2021a). The reason that spinosad did not perform as expected (Dively et al. 2020) is unlikely to be the result of the surfactant used as 2 different products were used in 2020 and 2021, and adjuvants were not a significant factor in onion thrips numbers in a previous study (Iglesias et al. 2021a). The sensitivity of onion thrips to spinetoram has been evaluated in New York due to concerns that thrips are developing resistance to this insecticide (Moretti et al. 2019). Despite a variation in LC_50_ levels among different field populations, New York growers reported that spinetoram performed as expected. This is supported by spray trial results, including our 2022 results which indicate that it remains effective in the field (Moretti et al. 2019). In Michigan, spinetoram has been used by growers extensively for the past >10 years so the lack of efficacy for spinosad could be due to an increase in resistance to spinosyn insecticides (Siegwart et al. 2015). It is possible that low levels of resistance are detected when applying the bioinsecticide formulation of this insecticide which may have lower longevity and thus efficacy in the field than the conventional version. Monitoring onion thrips’ susceptibility to spinosyns using appropriate bioassays should be a focus for geographic regions that rely on these insecticides for onion production (Yannuzzi et al. 2021).

Copper alone did not reduce the amount of necrotic tissue, but the amount of necrotic tissue was lower in spinosad + copper-treated and spinosad-treated plots in 2020. In the case of spinosad-treated plots, the average thrips numbers were similar to those in other treatments that did not reduce necrotic tissue. We did not count adult and immature thrips separately, but it is possible that spinosad was more effective against immature than adult stages, which has been observed for some bioinsecticides (Dively et al. 2020). Overall, copper did not contribute to reducing necrotic tissue and it is possible that the presence of onion thrips feeding reduced the efficacy of copper to control diseases (Stumpf et al. 2021). In 2021, the conventional treatment (CI + CF) had lower amounts of necrotic tissue relative to the other treatments. Leaf necrosis and thrips action thresholds increased in concert from low to high, in 2022, thus the measure of leaf necrosis better predicted our expected thrips performance than average thrips numbers. This may have been because we were measuring relatively small differences among treatments that may have been masked by the variability in insect numbers on plants but were captured in data that reflects cumulative season-long effects. In the future, it will be important to increase sample size and reduce variation.

The conventional insecticide programs significantly reduced the average number of thrips in both years below the action threshold, regardless of tank mixing with fungicides. However, chlorothalonil can interfere with insecticide efficacy in some instances (Nault et al. 2013). In 2022, thrips levels were numerically higher when insecticides were applied with rather than without a fungicide. In 2022, the conventional fungicide-only-treated plots significantly reduced necrotic tissue relative to the control, while the average numbers of thrips were similar to the untreated plots. This is likely due to the fungicides providing control against common leaf blight pathogens. The different thresholds we tested in 2022 did not significantly influence average thrips numbers; insect pressure overall was low in 2022 and our thresholds were relatively similar to each other; therefore, it was difficult to separate these treatments statistically. In the future, thresholds that span a larger range should be tested for their efficacy to determine how thrips and onion diseases could be managed together effectively.

In conclusion, our results suggest that biopesticides have low efficacy for controlling onion pests and their relatively high cost may hinder their adoption in large-scale agriculture. We were not able to conclusively determine in this study if tank mixing of fungicides and insecticides leads to reduced amounts of necrotic tissue, but this issue and the different action thresholds should be further tested in years when onion thrips pressure is high. In addition, using the recommended action threshold is likely to lead to significant savings for growers. In a year when onion thrips pressure was low (2022), 2 insecticide applications were as effective as 6 at managing onion thrips, indicating that a threshold-based management program could significantly contribute to reducing economic inputs and insecticide resistance risks (Leach et al. 2019). Indeed, onion growers who adopted threshold-based onion thrips management reduced their insecticide use by 1–4 applications leading to appreciable savings (Leach et al. 2019).

## Supplementary Material

toad201_suppl_Supplementary_Figures

## References

[CIT0001] Adesanya AW , Waters TD, Lavine MD, Walsh DB, Lavine LC, Zhu F. Multiple insecticide resistance in onion thrips populations from Western USA. Pestic Biochem Physiol. 2020:165:104553. 10.1016/j.pestbp.2020.104553. 32359535

[CIT0002] Anonymous. Government Accountability Office, management improvements needed to further promote integrated pest management (GAO Publication No. 01-815). Washington (D.C.): U.S. Government Printing Office; 2001.

[CIT0003] Bates D , Maechler M, Bolker B, Walker S. Fitting linear mixed-effects models using lme4. J Stat Softw. 2015:67(1):1–48.

[CIT0004] Bhangale GT , Joi MB. Role of thrips in the development of purple blotch of onion. J Maharashtra Agric Univ. 1983:8:299–300.

[CIT0005] Constancio N , Higgins D, Hausbeck M, Szendrei Z. Onion thrips (Thysanoptera: Thripidae) host plant preference and performance are mediated by a facultative plant pathogen of onion. Environ Entomol. 2022:51(6):1158–1165. 10.1093/ee/nvac086. 36351053

[CIT0006] Devi MS , Roy K. An integrated approach for management of *Thrips* tabaci Lindeman in rabi onion under Gangetic plains of West Bengal. Int J Curr Microbiol Appl Sci. 2018:7(07):2865–2877. 10.20546/ijcmas.2018.707.336.

[CIT0007] Diaz-Montano J , Fuchs M, Nault BA, Fail J, Shelton AM. Onion thrips (Thysanoptera: Thripidae): a global pest of increasing concern in onion. J Econ Entomol. 2011:104(1):1–13. 10.1603/ec10269. 21404832

[CIT0008] Dively GP , Patton T, Barranco L, Kulhanek K. Comparative efficacy of common active ingredients in organic insecticides against difficult to control insect pests. Insects. 2020:11(9):614–631. 10.3390/insects11090614. 32911857 PMC7565045

[CIT0009] Dutta B , Barman AK, Srinivasan R, Avci U, Ullman DE, Langston DB, Gitaitis RD. Transmission of *Pantoea ananatis* and *P. agglomerans*, causal agents of center rot of onion (*Allium cepa*), by onion thrips (*Thrips tabaci*) through feces. Phytopathology. 2014:104(8):812–819. 10.1094/PHYTO-07-13-0199-R. 24548212

[CIT0010] Fenibo EO , Ijoma GN, Matambo T. Biopesticides in sustainable agriculture: a critical sustainable development driver governed by green chemistry principles. Front Sustain Food Syst. 2021:5:1–6.

[CIT0011] Fournier F , Boivin G, Stewart RK. Effect of *Thrips tabaci* (Thysanoptera: Thripidae) on yellow onion yields and economic thresholds for its management. J Econ Entomol. 1995:88(5):1401–1407. 10.1093/jee/88.5.1401.

[CIT0012] Fronk L. Are you using biopesticides? PennState Extension. 2022 [accessed 2023 March]. https://extension.psu.edu/are-you-using-biopesticides.

[CIT0013] Gill HK , Garg H, Gill AK, Gillett-Kaufman JL, Nault BA. Onion thrips (Thysanoptera: Thripidae) biology, ecology, and management in onion production systems. J Integr Pest Manag. 2015:6(1):6. 10.1093/jipm/pmv006.

[CIT0014] Golec JR , Hoge B, Walgenbach JF. Effect of biopesticides on different *Tetranychus urticae* Koch (Acari: Tetranychidae) life stages. Crop Prot. 2020:128:105015. 10.1016/j.cropro.2019.105015.

[CIT0015] Grode A , Chen S, Walker ED, Szendrei Z. Onion thrips (Thysanoptera: Thripidae) feeding promotes infection by *Pantoea ananatis* in onion. J Econ Entomol. 2017:110(6):2301–2307. 10.1093/jee/tox273. 29112728 PMC6281329

[CIT0016] Grode AS , Brisco-McCann E, Wiriyajitsonboom P, Hausbeck MK, Szendrei Z. Managing onion thrips can limit bacterial stalk and leaf necrosis in Michigan onion fields. Plant Dis. 2019:103(5):938–943. 10.1094/PDIS-07-18-1271-RE. 30893026

[CIT0017] Hausbeck M , Werling B. Protect onions against Stemphylium leaf blight. Michigan State University Vegetable Extension; 2018. https://www.canr.msu.edu/news/protect-onions-against-stemphylium-leaf-blight.

[CIT0018] Hay F , Heck D, Sharma S, Klein A, Hoepting C, Pethybridge S. Stemphylium leaf blight of onion. Plant Heal Instr. 2022:22. 10.1094/PDIS-07-21-1587-RE 34798786

[CIT0019] Hothorn T , Bretz F, Westfall P. Simultaneous inference in general parametric models. Biom J. 2023:50(3):346–363. 10.1002/bimj.200810425. 18481363

[CIT0020] Iglesias L , Groves RL, Bradford B, Harding RS, Nault BA. Evaluating combinations of bioinsecticides and adjuvants for managing *Thrips tabaci* (Thysanoptera: Thripidae) in onion production systems. Crop Prot. 2021a:142:105527. 10.1016/j.cropro.2020.105527.

[CIT0021] Iglesias L , Havey MJ, Nault BA. Management of onion thrips (*Thrips tabaci*) in organic onion production using multiple IPM tactics. Insects. 2021b:12(3):207. 10.3390/insects12030207. 33804399 PMC8000123

[CIT0022] Leach A , Reiners S, Fuchs M, Nault B. Evaluating integrated pest management tactics for onion thrips and pathogens they transmit to onion. Agric Ecosyst Environ. 2017:250:89–101. 10.1016/j.agee.2017.08.031.

[CIT0023] Leach AB , Hoepting CA, Nault BA. Grower adoption of insecticide resistance management practices increase with extension-based program. Pest Manag Sci. 2019:75(2):515–526. 10.1002/ps.5150. 30047237

[CIT0024] Leach A , Hay F, Harding R, Damann KC, Nault B. Relationship between onion thrips (*Thrips tabaci*) and *Stemphylium vesicarium* in the development of Stemphylium leaf blight in onion. Ann Appl Biol. 2020:176(1):55–64. 10.1111/aab.12558.

[CIT0025] Lewis T. Feeding, flight and dispersal in thrips Towar Underst Thysanoptera. Proc Int Conf Thrips. 1991:63–70.

[CIT0026] Martin NA , Workman PJ, Butler RC. Insecticide resistance in onion thrips (*Thrips tabaci*) (Thysanoptera: Thripidae). NZ J Crop Hort Sci. 2003:31(2):99–106. 10.1080/01140671.2003.9514242.

[CIT0027] McKenzie CL , Cartwright B, Miller ME, Edelson JV. Injury to onions by *Thrips tabaci* (Thysanoptera: Thripidae) and its role in the development of purple blotch. Environ Entomol. 1993:22(6):1266–1277. 10.1093/ee/22.6.1266

[CIT0028] Moretti EA , Harding RS, Scott JG, Nault BA. Monitoring onion thrips (Thysanoptera: Thripidae) susceptibility to spinetoram in New York onion fields. J Econ Entomol. 2019:112(3):1493–1497. 10.1093/jee/toz032. 30805650

[CIT0029] MSU Enviroweather: Michigan State University. 2023 [accessed 2023 March]. https://enviroweather.msu.edu/.

[CIT0030] Nault BA , Lou Hessney M. Onion thrips control in onion. Arthropod Manag Tests. 2005:31:1–2.

[CIT0031] Nault BA , Shelton AM. Impact of insecticide efficacy on developing action thresholds for pest management: a case study of onion thrips (Thysanoptera: Thripidae) on onion. J Econ Entomol. 2010:103(4):1315–1326. 10.1603/ec10096. 20857743

[CIT0032] Nault BA , Hsu CL, Hoepting CA. Consequences of co-applying insecticides and fungicides for managing *Thrips tabaci* (Thysanoptera: Thripidae) on onion. Pest Manag Sci. 2013:69(7):841–849. 10.1002/ps.3444. 23197362

[CIT0033] Pimentel D. Environmental and economic costs of the application of pesticides primarily in the United States. Environ Dev Sustain. 2005:7(2):229–252. 10.1007/s10668-005-7314-2.

[CIT0034] R Core Team. R: A language and environment for statistical computing. Vienna (Austria): R Foundation for Statistical Computing; 2023. https://www.R-project.org/.

[CIT0035] Reddy PP. Sustainable crop protection under protected cultivation. Singapore: Springer; 2016.

[CIT0036] Rodriguez-Salamanca LM , Enzenbacher TB, Derie ML, du Toit LJ, Feng C, Hausbeck MK. First report of *Colletotrichum coccodes* causing leaf and neck anthracnose in onions (*Allium cepa*) in Michigan and the United States. Plant Dis. 2012:96:769–769. 10.1094/PDIS-01-12-0022-PDN 30727550

[CIT0037] Rolim GS , Plata-Rueda A, Martínez LC, Ribeiro GT, Serrão JE, Zanuncio JC. Side effects of *Bacillus thuringiensis* on the parasitoid *Palmistichus elaeisis* (Hymenoptera: Eulophidae). Ecotoxicol Environ Saf. 2020:189:109978. 10.1016/j.ecoenv.2019.109978. 31761554

[CIT0038] Serrão JE , Plata-Rueda A, Martínez LC, Zanuncio JC. Side-effects of pesticides on non-target insects in agriculture: a mini-review. Die Naturwissenschaften. 2022:109(2):17. 10.1007/s00114-022-01788-8. 35138481

[CIT0039] Shelton AM , Zhao JZ, Nault BA, Plate J, Musser FR, Larentzaki E. Patterns of insecticide resistance in onion thrips (Thysanoptera: Thripidae) in onion fields in New York. J Econ Entomol. 2006:99(5):1798–1804. 10.1093/jee/99.5.1798 17066815

[CIT0040] Siegwart M , Graillot B, Lopez CB, Besse S, Bardin M, Nicot PC, Lopez-Ferber M. Resistance to bio-insecticides or how to enhance their sustainability: a review. Front Plant Sci. 2015:6:1–19. 26150820 10.3389/fpls.2015.00381 PMC4472983

[CIT0041] Silva WM , Martínez LC, Plata-Rueda A, Serrão JE, Zanuncio JC. Respiration, predatory behavior and prey consumption by *Podisus nigrispinus* (Heteroptera: Pentatomidae) nymphs exposed to some insecticides. Chemosphere. 2020:261:127720. 10.1016/j.chemosphere.2020.127720. 32721693

[CIT0042] Sparks TC. Insecticide discovery: an evaluation and analysis. Pestic Biochem Physiol. 2013:107(1):8–17. 10.1016/j.pestbp.2013.05.012 25149229

[CIT0043] Stumpf S , Leach L, Srinivasan R, Coolong T, Gitaitis R, Dutta B. Foliar chemical protection against *Pantoea ananatis* in onion is negated by thrips feeding. Phytopathology. 2021:111(2):258–267. 10.1094/PHYTO-05-20-0163-R. 32748732

[CIT0044] Ternest JJ , Ingwell LL, Foster RE, Kaplan I, Nielsen A. Comparing prophylactic versus threshold-based insecticide programs for striped cucumber beetle (Coleoptera: Chrysomelidae) management in watermelon. J Econ Entomol. 2020:113:872–881. 31901943 10.1093/jee/toz346 PMC7136195

[CIT0045] United States Department of Agriculture. Economic Research Service. National Organic Program Standards Code 205.206 (e). United States Department of Agriculture; 2023. https://www.ams.usda.gov/rules-regulations/organic.

[CIT0046] Woudenberg JHC , Hanse B, van Leeuwen GCM, Groenewald JZ, Crous PW. Stemphylium revisited. Stud Mycol. 2017:87:77–103. 10.1016/j.simyco.2017.06.001 28663603 PMC5480992

[CIT0047] Yadav M , Prasad R, Kumar P, Pandey C. A review on onion thrips and their management of bulb crops. J Pharmacogn Phytochem Ctry. 2018:7(1S):891–896.

[CIT0048] Yannuzzi IM , Moretti EA, Nault BA. Comparison of bioassays used to determine onion thrips (Thysanoptera: Thripidae) susceptibility to spinetoram. J Econ Entomol. 2021:114(5):2236–2240. 10.1093/jee/toab136 34289041

