# Supplementary material for: Managing insect and plant pathogen pests with organic and conventional pesticides in onions
Source: J Econ Entomol. 2023 Nov 1;116(6):2116–23. doi: 10.1093/jee/toad201 (PMC10711546; doi:10.1093/jee/toad201)
Supplement: toad201_suppl_Supplementary_Figures [file toad201_suppl_supplementary_figures.docx]

**Supplemental material**

**Area under the disease progress curve (AUDPC)**

**Methods**

To compare how the pesticide programs affected the amount of necrotic onion tissue, we calculated the area under the disease progress curve (AUDPC) for each year. The AUDPC is used to combine multiple observations of the necrotic tissue progression throughout the season to compare across treatments using a single value (Jeger and Viljanen-Rollinson 2000). The AUDPC values were compared with an ANOVA with treatment as the fixed factor using the ‘lme4’ package. The ‘multcomp’ package was used for separating treatment means (Tukey’s HSD test, α = 0.05). The AUDPC was calculated using the ‘agricolae’ package (v1.3-5; de Mendiburu, 2022) in R (R Core Team 2023).

**Results**

In 2020, there was a significant effect of treatment on disease progression (*F* = 8.03, df = 5, 18, *P* < 0.01). Compared to the control, treatments with spinosad (t-value = 4.19, df = 6, *P* < 0.01) and treatments with spinosad + copper (t-value = 4.48, df = 6, *P* < 0.01), significantly reduced disease progression throughout the season.

There was a significant effect of treatment on disease progression (*F* = 9.96, df = 7, 24, *P* < 0.01) in 2021. Compared to the control, the conventional insecticide + copper (CI + copper) (t-value = 4.79, df = 8, *P* < 0.01) and the combined conventional insecticide and conventional fungicide program (CI + CF) (t-value = 6.36, df = 8, *P* < 0.01) significantly reduced disease progression throughout the season. There was no difference in disease progression between spinosad and conventional insecticide + copper spray programs, but the fully conventional (insecticide + fungicide) spray program significantly reduced disease progression compared to the spinosad treatment (t-value = 3.23, df = 8, *P* = 0.016). There was no significant difference in disease progression in the conventional insecticide + copper and the fully conventional program.

In 2022, treatment significantly affected disease progression (*F* = 4.3, df = 7, 24, *P* < 0.01). Compared to the control treatment, the low action threshold + fungicide significantly reduced disease progression throughout the season (t-value = 4.15, df = 14, *P* < 0.01). The low action threshold + fungicide also significantly reduced disease progression compared to the moderate action threshold without fungicide (t-value = -3.43, df = 14, *P* = 0.04) and the high action threshold without fungicide (t-value = -4.19, df = 14, *P* < 0.01).

**Figures and figure captions**

**
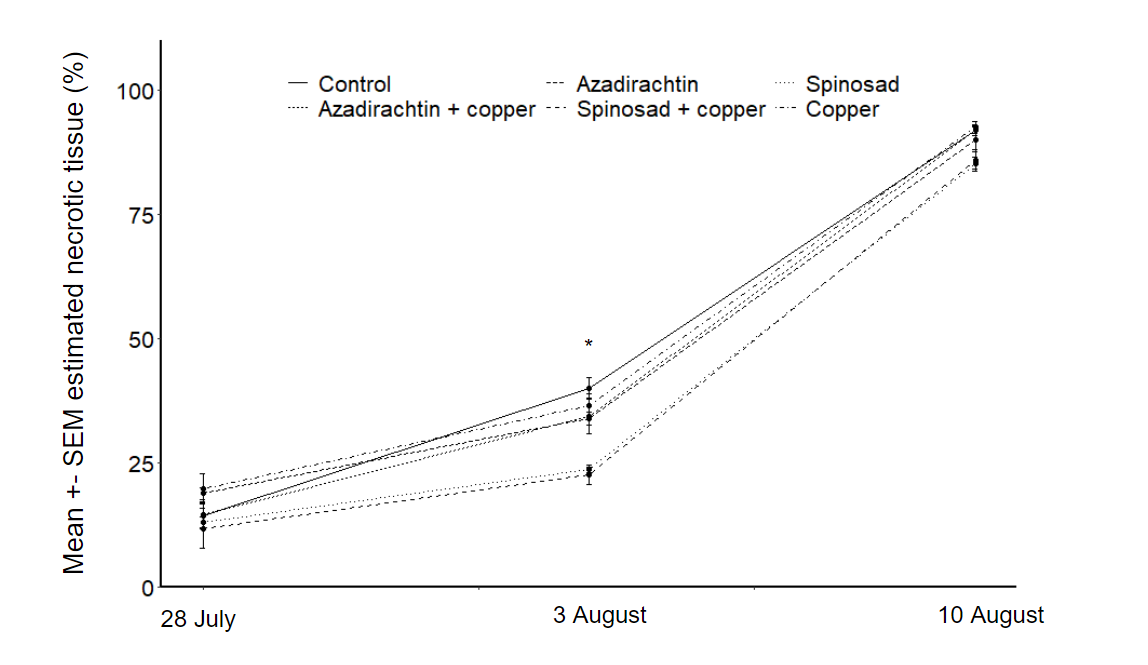
**

Supplementary Figure 1. In 2020, we tested 6 different pesticide programs in an onion field. The amount of necrotic tissue per plot was visually estimated 3 times throughout the growing season. On July 28^th^, less than 25% of the plot had necrotic tissue, on August 10^th^, approximately 80% of the plots were necrotic. Asterisks represent significant differences among treatments, within a date (Tukey’s HSD, α = 0.05).


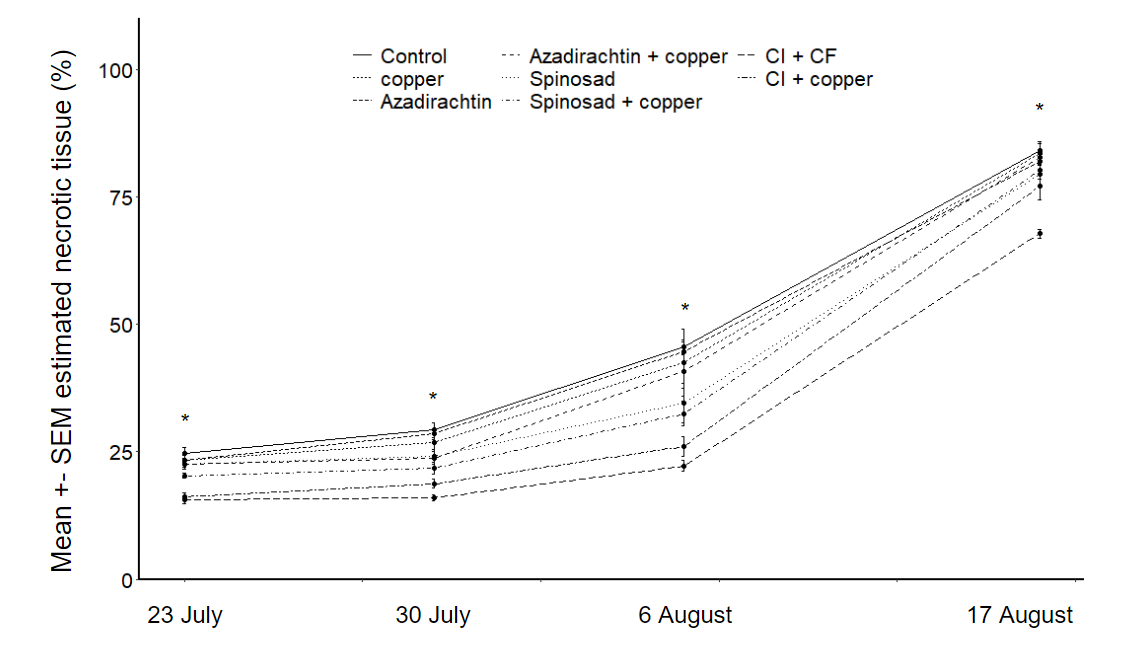


Supplementary Figure 2. In 2021, we tested 8 different pesticide programs in an onion field. Treatments labeled CI + copper represents the conventional insecticide program + copper fungicide, while the CI + CF treatment is the conventional insecticide program paired with a conventional fungicide program. The amount of necrotic tissue per plot was visually estimated 4 times at the end of the growing season. Asterisks represent significant differences among treatments, within dates (Tukey’s HSD, α = 0.05).


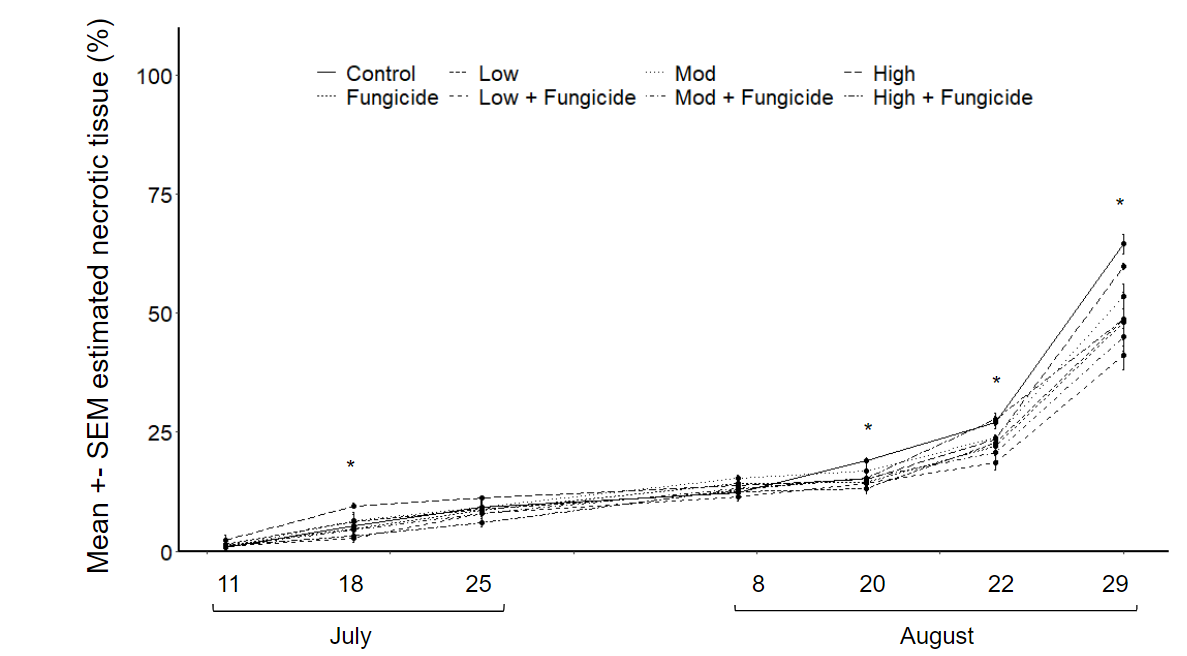


Supplementary Figure 3. In 2022, we tested 8 different pesticide programs in an onion field, these included a combination of pesticides applied when an onion thrips action threshold (Low = 0.5 thrips/leaf; Mod = 0.6 thrips/leaf; High = 1.0 thrips/leaf) was reached. The amount of necrotic tissue per plot was visually estimated weekly throughout the growing season. Asterisks represent significant differences among treatments, within dates (Tukey’s HSD, α = 0.05).

**References**

de Mendiburu, F. 2022. Agricolae: Statistical Procedures for Agricultural Research. R package version 1.3-5, [https://CRAN](about:blank).R-project.org/package=agricolae

Jeger, M. J., and S. L. H. Viljanen-Rollinson. 2001. The use of the area under the disease-progress curve (AUDPC) to assess quantitative disease resistance in crop cultivars. Theor. Appl. Genet. 102: 32–40.

R Core Team. 2023. *R: A language and environment for statistical computing.* R Foundation for Statistical Computing. Vienna, Austria. [https://www.R-project.org/](about:blank)
